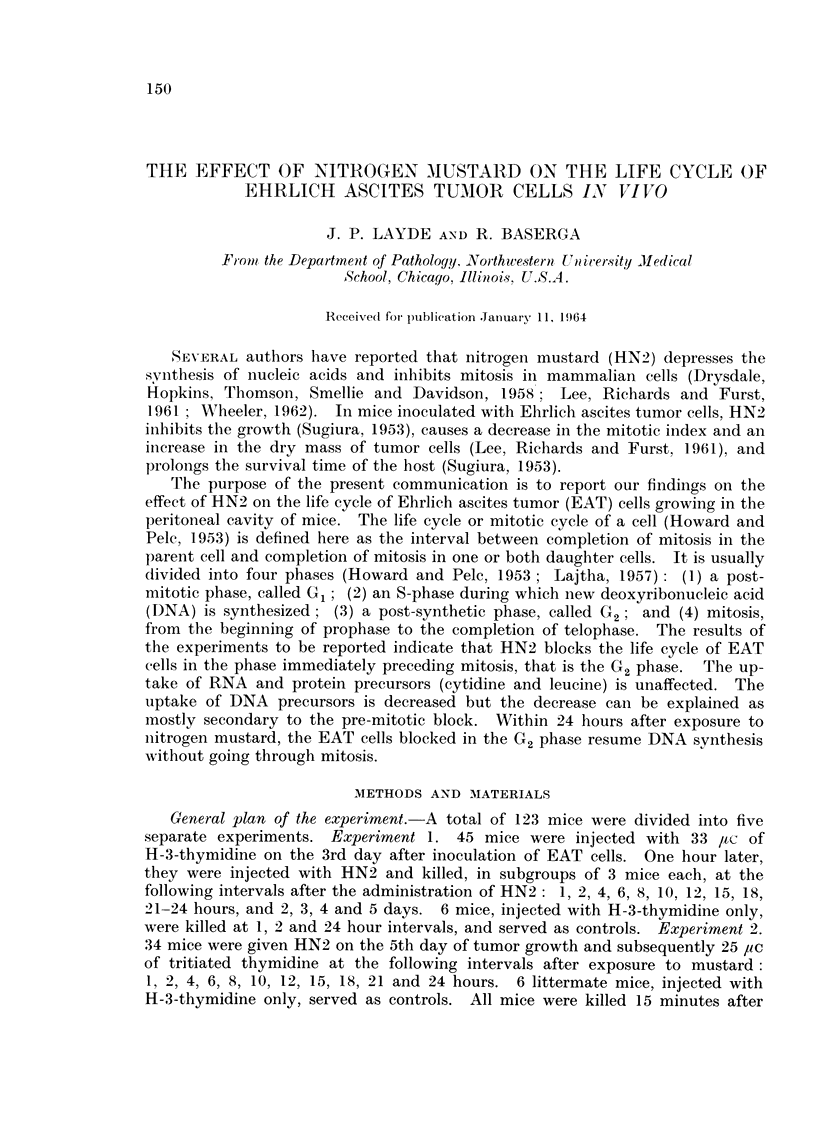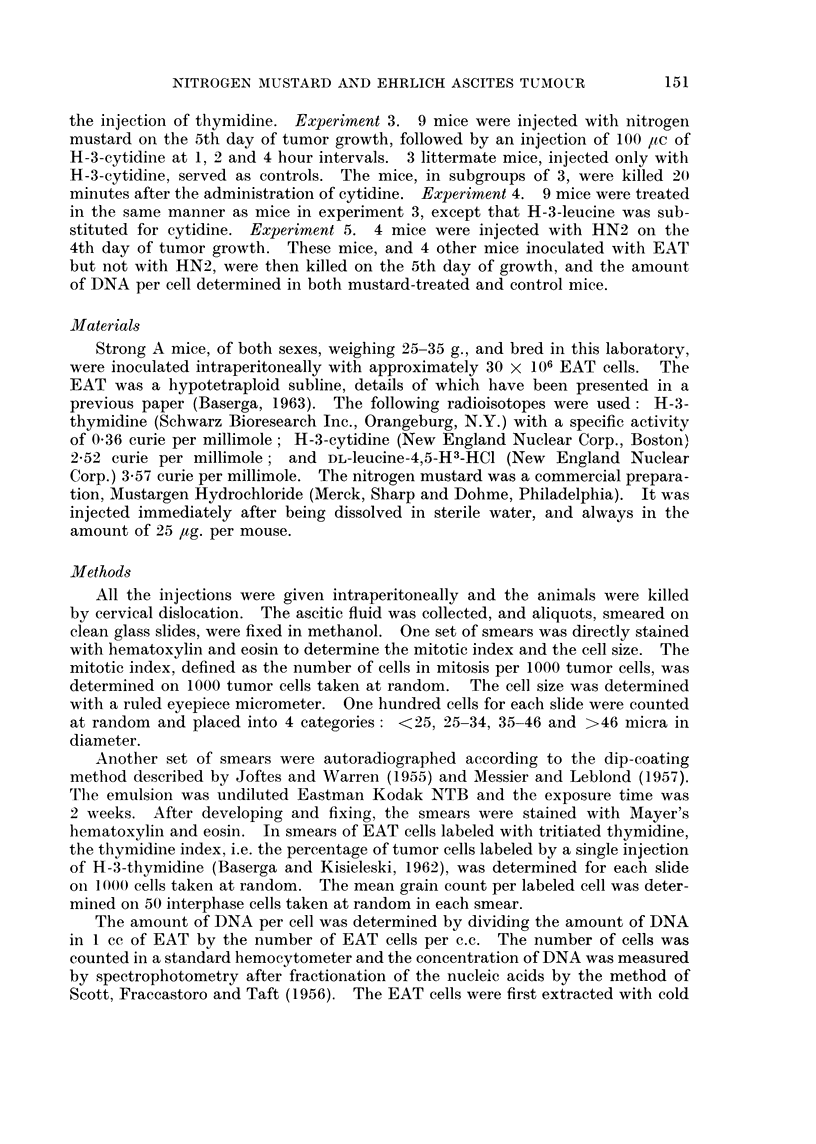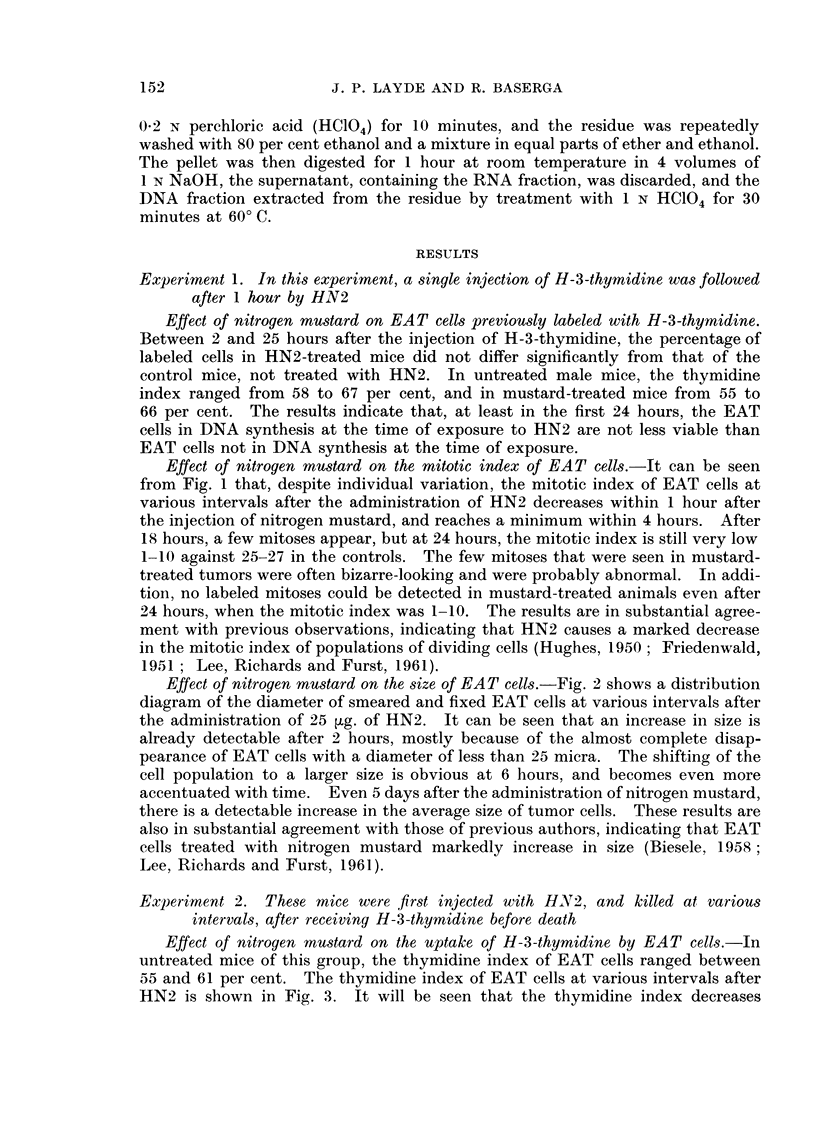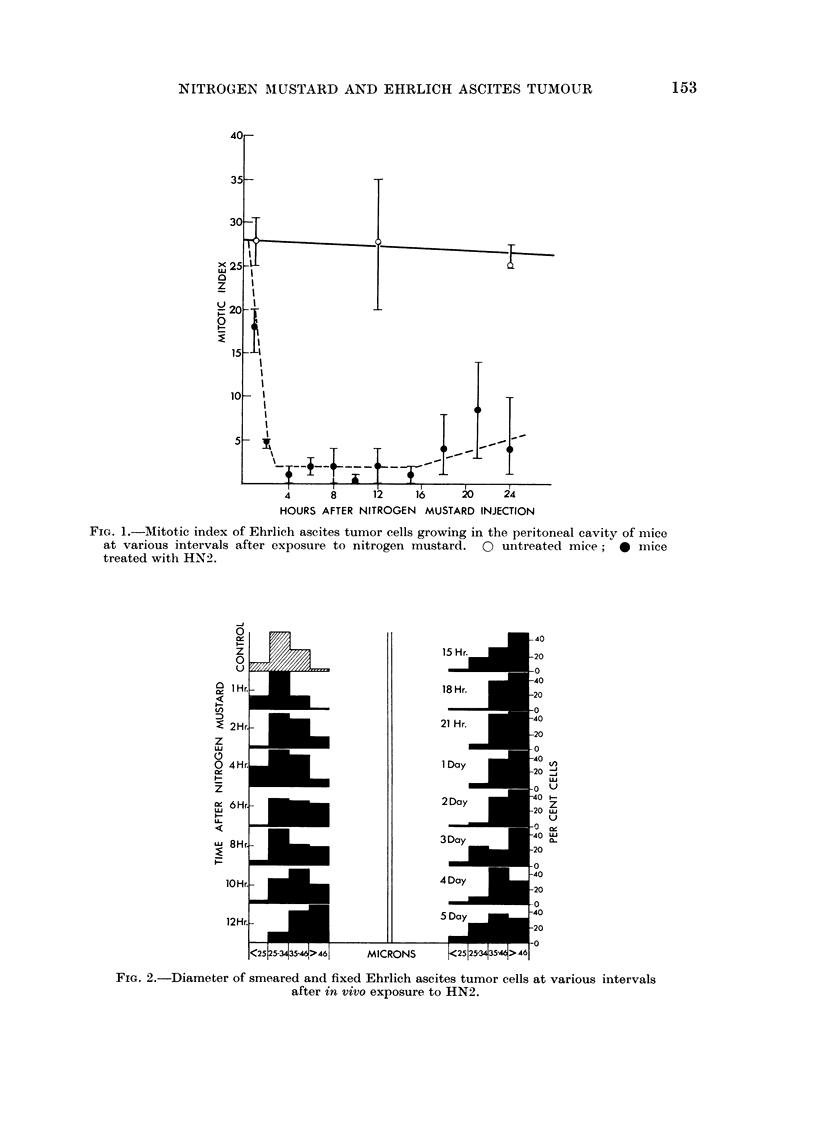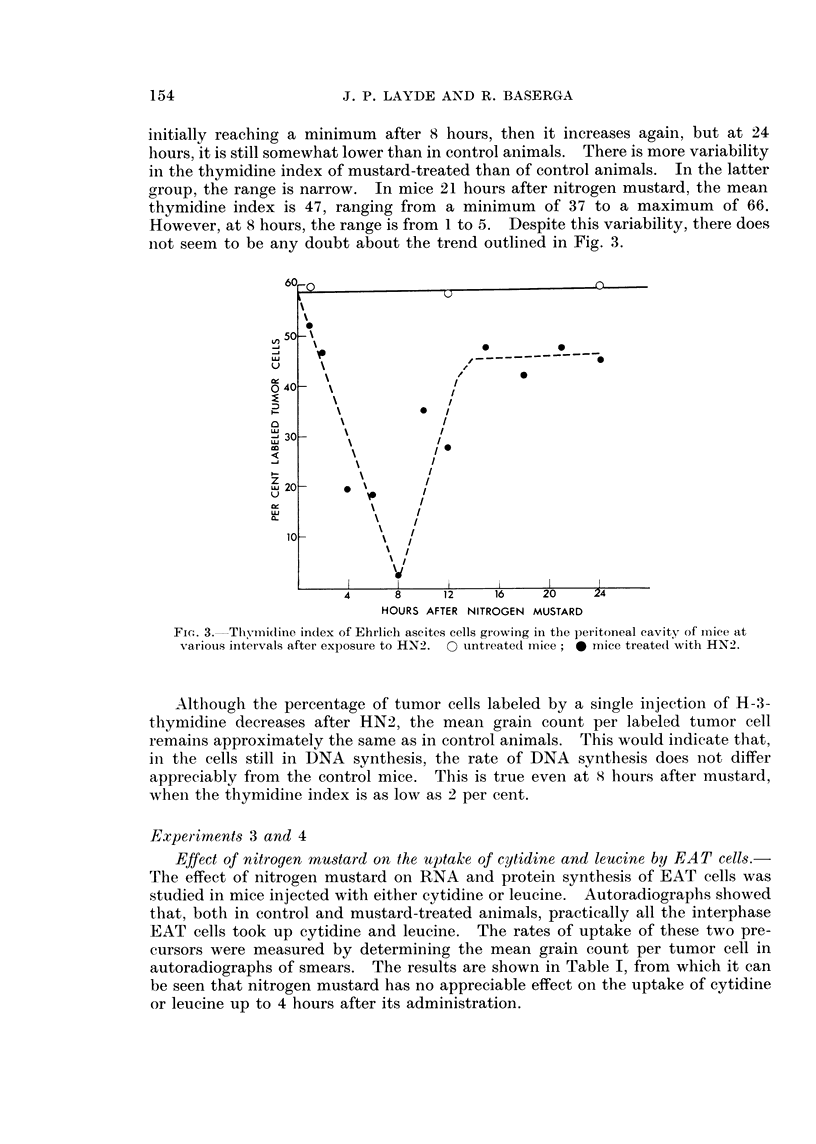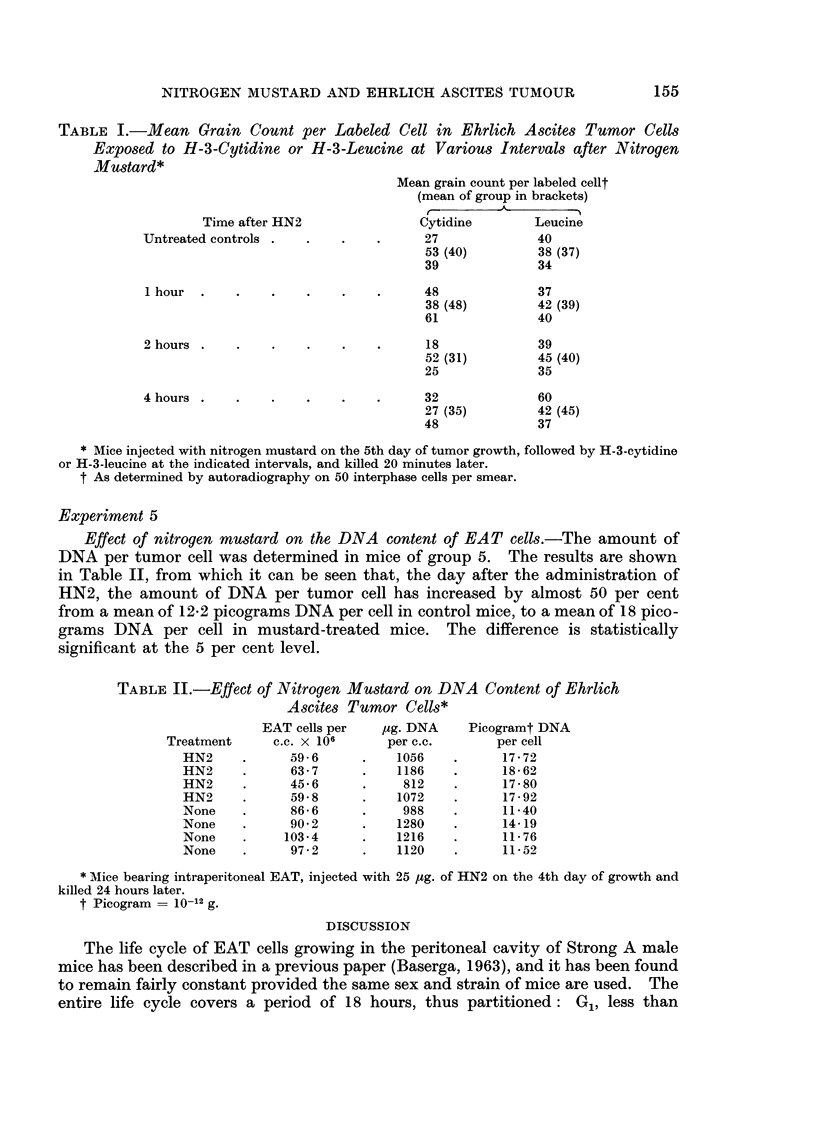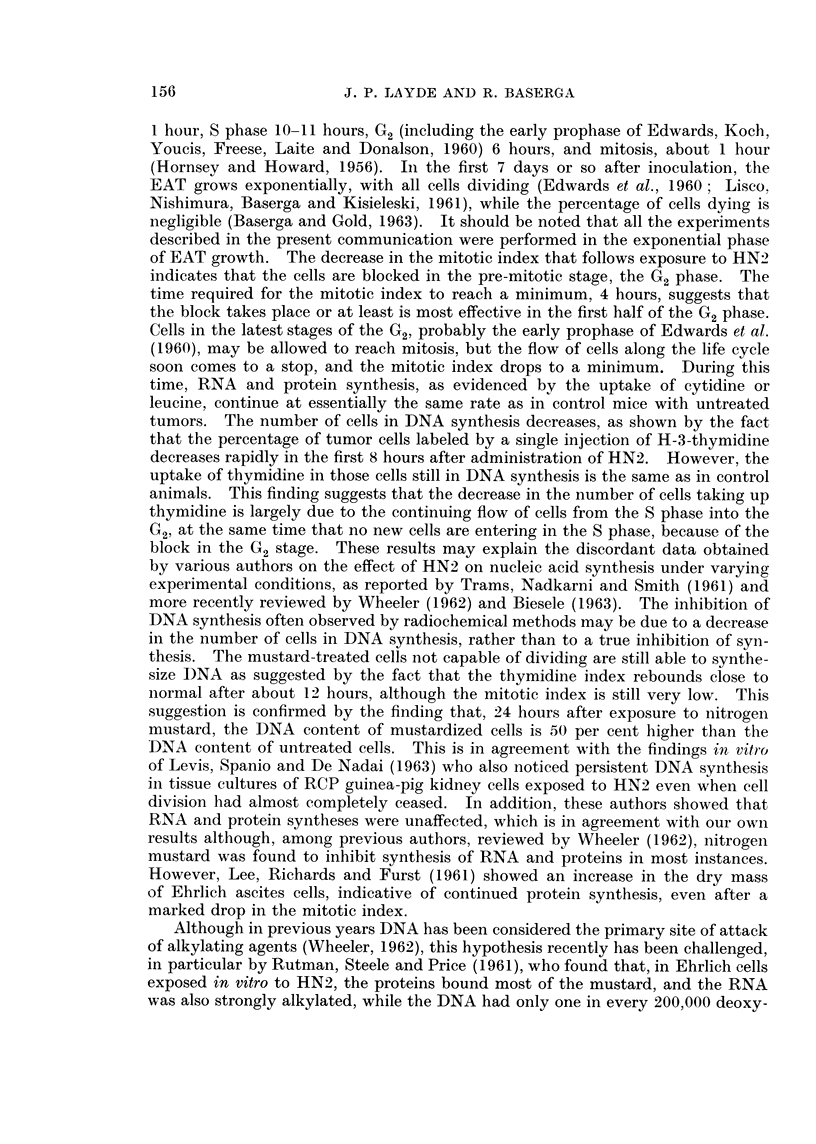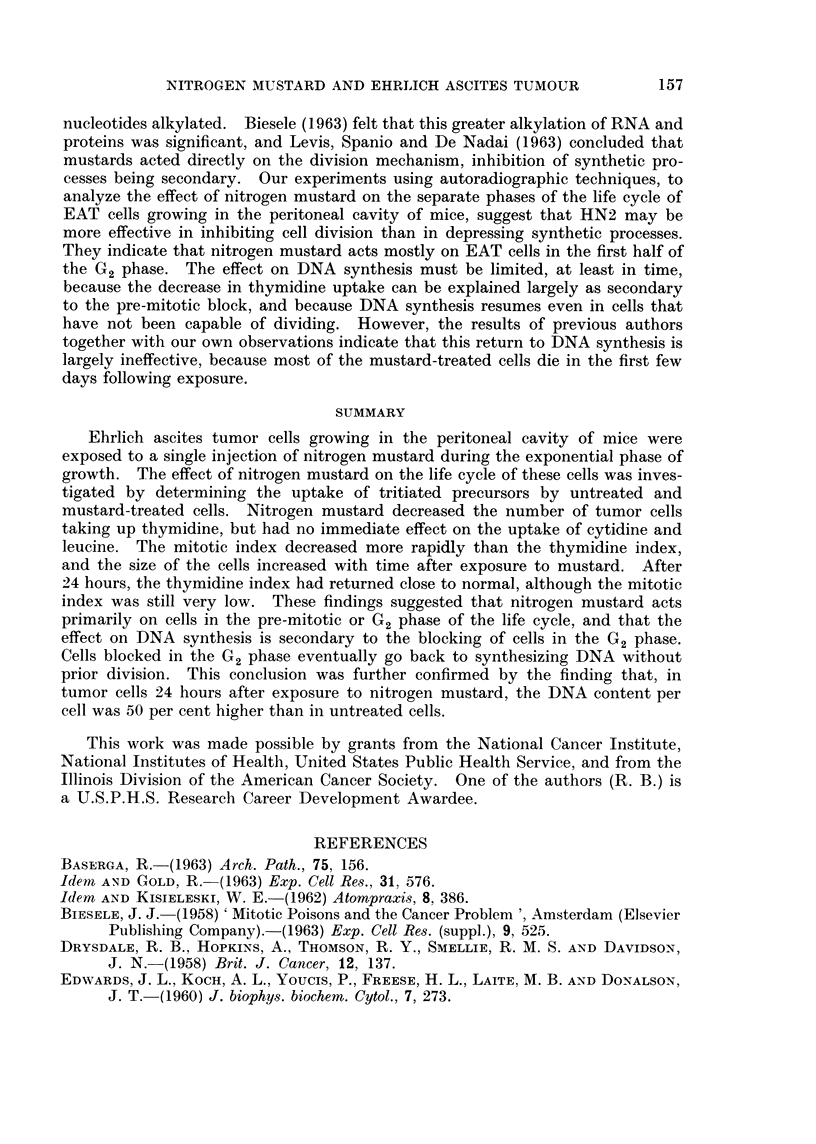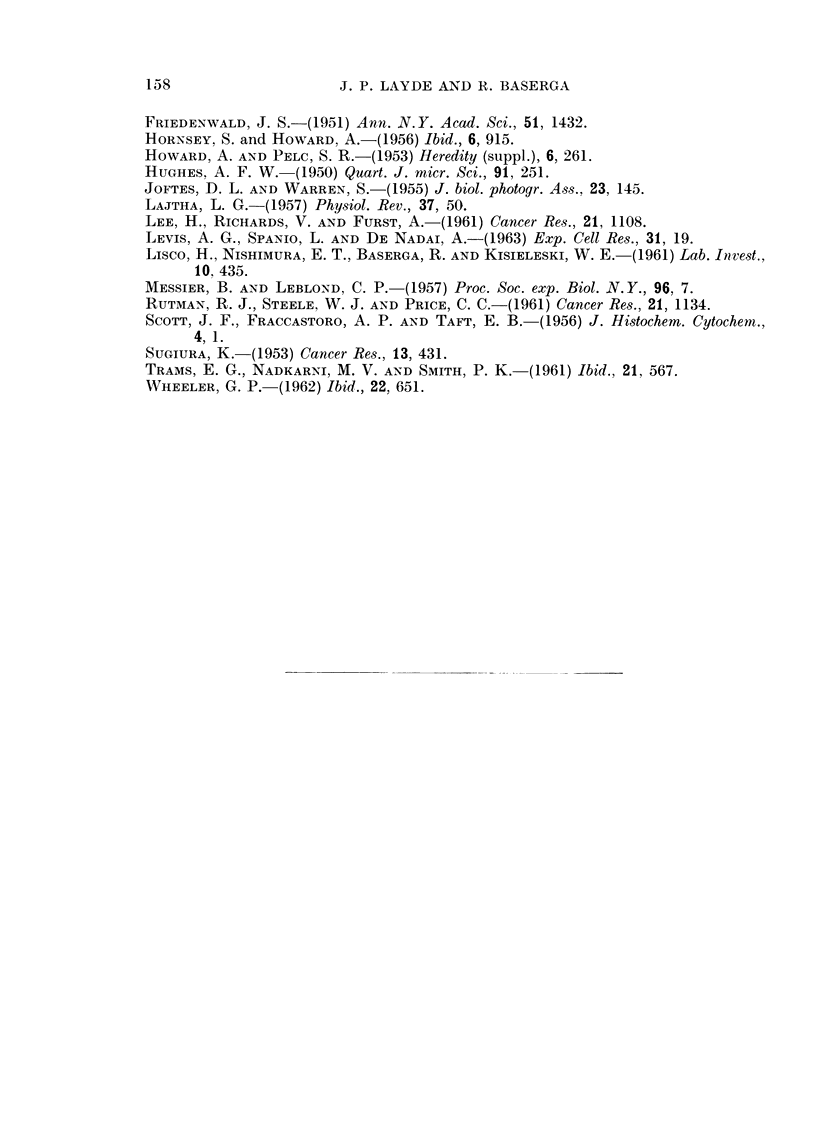# The Effect of Nitrogen Mustard on the Life Cycle of Ehrlich Ascites Tumour Cells In Vivo

**DOI:** 10.1038/bjc.1964.17

**Published:** 1964-03

**Authors:** J. P. Layde, R. Baserga


### THE EFFECT OF NITROGEN MUSTARD ON THE LIFE CYCLE OF EHRLICH ASCITES TUMOR CELLS *IN VIVO*

J. P. LAYDE AND R. BASERGA

*From the Department of Pathology, Northwestern University Medical School, Chicago, Illinois, U.S.A.*

Received for publication January 11, 1964

SEVERAL authors have reported that nitrogen mustard (HN2) depresses the synthesis of nucleic acids and inhibits mitosis in mammalian cells (Drysdale, Hopkins, Thomson, Smellie and Davidson, 1958; Lee, Richards and Furst, 1961; Wheeler, 1962). In mice inoculated with Ehrlich ascites tumor cells, HN2 inhibits the growth (Sugiura, 1953), causes a decrease in the mitotic index and an increase in the dry mass of tumor cells (Lee, Richards and Furst, 1961), and prolongs the survival time of the host (Sugiura, 1953).

The purpose of the present communication is to report our findings on the effect of HN2 on the life cycle of Ehrlich ascites tumor (EAT) cells growing in the peritoneal cavity of mice. The life cycle or mitotic cycle of a cell (Howard and Pelc, 1953) is defined here as the interval between completion of mitosis in the parent cell and completion of mitosis in one or both daughter cells. It is usually divided into four phases (Howard and Pelc, 1953; Lajtha, 1957): (1) a post-mitotic phase, called $G_1$; (2) an S-phase during which new deoxyribonucleic acid (DNA) is synthesized; (3) a post-synthetic phase, called $G_2$; and (4) mitosis, from the beginning of prophase to the completion of telophase. The results of the experiments to be reported indicate that HN2 blocks the life cycle of EAT cells in the phase immediately preceding mitosis, that is the $G_2$ phase. The uptake of RNA and protein precursors (cytidine and leucine) is unaffected. The uptake of DNA precursors is decreased but the decrease can be explained as mostly secondary to the pre-mitotic block. Within 24 hours after exposure to nitrogen mustard, the EAT cells blocked in the $G_2$ phase resume DNA synthesis without going through mitosis.

### METHODS AND MATERIALS

*General plan of the experiment.*—A total of 123 mice were divided into five separate experiments. *Experiment* 1. 45 mice were injected with 33 $\mu c$ of H-3-thymidine on the 3rd day after inoculation of EAT cells. One hour later, they were injected with HN2 and killed, in subgroups of 3 mice each, at the following intervals after the administration of HN2: 1, 2, 4, 6, 8, 10, 12, 15, 18, 21–24 hours, and 2, 3, 4 and 5 days. 6 mice, injected with H-3-thymidine only, were killed at 1, 2 and 24 hour intervals, and served as controls. *Experiment* 2. 34 mice were given HN2 on the 5th day of tumor growth and subsequently 25 $\mu c$ of tritiated thymidine at the following intervals after exposure to mustard: 1, 2, 4, 6, 8, 10, 12, 15, 18, 21 and 24 hours. 6 littermate mice, injected with H-3-thymidine only, served as controls. All mice were killed 15 minutes after the injection of thymidine. *Experiment* 3. 9 mice were injected with nitrogen mustard on the 5th day of tumor growth, followed by an injection of 100 $\mu c$ of H-3-cytidine at 1, 2 and 4 hour intervals. 3 littermate mice, injected only with H-3-cytidine, served as controls. The mice, in subgroups of 3, were killed 20 minutes after the administration of cytidine. *Experiment* 4. 9 mice were treated in the same manner as mice in experiment 3, except that H-3-leucine was substituted for cytidine. *Experiment* 5. 4 mice were injected with HN2 on the 4th day of tumor growth. These mice, and 4 other mice inoculated with EAT but not with HN2, were then killed on the 5th day of growth, and the amount of DNA per cell determined in both mustard-treated and control mice.

*Materials*

Strong A mice, of both sexes, weighing 25–35 g., and bred in this laboratory, were inoculated intraperitoneally with approximately $30 \times 10^6$ EAT cells. The EAT was a hypotetraploid subline, details of which have been presented in a previous paper (Baserga, 1963). The following radioisotopes were used: H-3-thymidine (Schwarz Bioresearch Inc., Orangeburg, N.Y.) with a specific activity of 0·36 curie per millimole; H-3-cytidine (New England Nuclear Corp., Boston) 2·52 curie per millimole; and DL-leucine-4,5-$H^3$-HCl (New England Nuclear Corp.) 3·57 curie per millimole. The nitrogen mustard was a commercial preparation, Mustargen Hydrochloride (Merck, Sharp and Dohme, Philadelphia). It was injected immediately after being dissolved in sterile water, and always in the amount of 25 $\mu$g. per mouse.

*Methods*

All the injections were given intraperitoneally and the animals were killed by cervical dislocation. The ascitic fluid was collected, and aliquots, smeared on clean glass slides, were fixed in methanol. One set of smears was directly stained with hematoxylin and eosin to determine the mitotic index and the cell size. The mitotic index, defined as the number of cells in mitosis per 1000 tumor cells, was determined on 1000 tumor cells taken at random. The cell size was determined with a ruled eyepiece micrometer. One hundred cells for each slide were counted at random and placed into 4 categories: $<25$, 25–34, 35–46 and $>46$ micra in diameter.

Another set of smears were autoradiographed according to the dip-coating method described by Joftes and Warren (1955) and Messier and Leblond (1957). The emulsion was undiluted Eastman Kodak NTB and the exposure time was 2 weeks. After developing and fixing, the smears were stained with Mayer's hematoxylin and eosin. In smears of EAT cells labeled with tritiated thymidine, the thymidine index, i.e. the percentage of tumor cells labeled by a single injection of H-3-thymidine (Baserga and Kisieleski, 1962), was determined for each slide on 1000 cells taken at random. The mean grain count per labeled cell was determined on 50 interphase cells taken at random in each smear.

The amount of DNA per cell was determined by dividing the amount of DNA in 1 cc of EAT by the number of EAT cells per c.c. The number of cells was counted in a standard hemocytometer and the concentration of DNA was measured by spectrophotometry after fractionation of the nucleic acids by the method of Scott, Fraccastoro and Taft (1956). The EAT cells were first extracted with cold

0·2 N perchloric acid ($HClO_4$) for 10 minutes, and the residue was repeatedly washed with 80 per cent ethanol and a mixture in equal parts of ether and ethanol. The pellet was then digested for 1 hour at room temperature in 4 volumes of 1 N NaOH, the supernatant, containing the RNA fraction, was discarded, and the DNA fraction extracted from the residue by treatment with 1 N $HClO_4$ for 30 minutes at 60° C.

### RESULTS

*Experiment* 1. *In this experiment, a single injection of H-3-thymidine was followed after* 1 *hour by HN*2

*Effect of nitrogen mustard on EAT cells previously labeled with H-3-thymidine.* Between 2 and 25 hours after the injection of H-3-thymidine, the percentage of labeled cells in HN2-treated mice did not differ significantly from that of the control mice, not treated with HN2. In untreated male mice, the thymidine index ranged from 58 to 67 per cent, and in mustard-treated mice from 55 to 66 per cent. The results indicate that, at least in the first 24 hours, the EAT cells in DNA synthesis at the time of exposure to HN2 are not less viable than EAT cells not in DNA synthesis at the time of exposure.

*Effect of nitrogen mustard on the mitotic index of EAT cells.*—It can be seen from Fig. 1 that, despite individual variation, the mitotic index of EAT cells at various intervals after the administration of HN2 decreases within 1 hour after the injection of nitrogen mustard, and reaches a minimum within 4 hours. After 18 hours, a few mitoses appear, but at 24 hours, the mitotic index is still very low 1–10 against 25–27 in the controls. The few mitoses that were seen in mustard-treated tumors were often bizarre-looking and were probably abnormal. In addition, no labeled mitoses could be detected in mustard-treated animals even after 24 hours, when the mitotic index was 1–10. The results are in substantial agreement with previous observations, indicating that HN2 causes a marked decrease in the mitotic index of populations of dividing cells (Hughes, 1950 ; Friedenwald, 1951 ; Lee, Richards and Furst, 1961).

*Effect of nitrogen mustard on the size of EAT cells.*—Fig. 2 shows a distribution diagram of the diameter of smeared and fixed EAT cells at various intervals after the administration of 25 μg. of HN2. It can be seen that an increase in size is already detectable after 2 hours, mostly because of the almost complete disappearance of EAT cells with a diameter of less than 25 micra. The shifting of the cell population to a larger size is obvious at 6 hours, and becomes even more accentuated with time. Even 5 days after the administration of nitrogen mustard, there is a detectable increase in the average size of tumor cells. These results are also in substantial agreement with those of previous authors, indicating that EAT cells treated with nitrogen mustard markedly increase in size (Biesele, 1958 ; Lee, Richards and Furst, 1961).

*Experiment* 2. *These mice were first injected with HN*2, *and killed at various intervals, after receiving H-*3*-thymidine before death*

*Effect of nitrogen mustard on the uptake of H-3-thymidine by EAT cells.*—In untreated mice of this group, the thymidine index of EAT cells ranged between 55 and 61 per cent. The thymidine index of EAT cells at various intervals after HN2 is shown in Fig. 3. It will be seen that the thymidine index decreases

FIG. 1.—Mitotic index of Ehrlich ascites tumor cells growing in the peritoneal cavity of mice at various intervals after exposure to nitrogen mustard. ○ untreated mice ; ● mice treated with HN2.

FIG. 2.—Diameter of smeared and fixed Ehrlich ascites tumor cells at various intervals after *in vivo* exposure to HN2.

initially reaching a minimum after 8 hours, then it increases again, but at 24 hours, it is still somewhat lower than in control animals. There is more variability in the thymidine index of mustard-treated than of control animals. In the latter group, the range is narrow. In mice 21 hours after nitrogen mustard, the mean thymidine index is 47, ranging from a minimum of 37 to a maximum of 66. However, at 8 hours, the range is from 1 to 5. Despite this variability, there does not seem to be any doubt about the trend outlined in Fig. 3.

FIG. 3.—Thymidine index of Ehrlich ascites cells growing in the peritoneal cavity of mice at various intervals after exposure to HN2. ○ untreated mice ; ● mice treated with HN2.

Although the percentage of tumor cells labeled by a single injection of H-3-thymidine decreases after HN2, the mean grain count per labeled tumor cell remains approximately the same as in control animals. This would indicate that, in the cells still in DNA synthesis, the rate of DNA synthesis does not differ appreciably from the control mice. This is true even at 8 hours after mustard, when the thymidine index is as low as 2 per cent.

*Experiments* 3 *and* 4

*Effect of nitrogen mustard on the uptake of cytidine and leucine by EAT cells.*—The effect of nitrogen mustard on RNA and protein synthesis of EAT cells was studied in mice injected with either cytidine or leucine. Autoradiographs showed that, both in control and mustard-treated animals, practically all the interphase EAT cells took up cytidine and leucine. The rates of uptake of these two precursors were measured by determining the mean grain count per tumor cell in autoradiographs of smears. The results are shown in Table I, from which it can be seen that nitrogen mustard has no appreciable effect on the uptake of cytidine or leucine up to 4 hours after its administration.

TABLE I.—*Mean Grain Count per Labeled Cell in Ehrlich Ascites Tumor Cells Exposed to H-3-Cytidine or H-3-Leucine at Various Intervals after Nitrogen Mustard**

| Time after HN2 | Mean grain count per labeled cell† (mean of group in brackets) | |
|---|---|---|
| | Cytidine | Leucine |
| Untreated controls | 27 | 40 |
| | 53 (40) | 38 (37) |
| | 39 | 34 |
| 1 hour | 48 | 37 |
| | 38 (48) | 42 (39) |
| | 61 | 40 |
| 2 hours | 18 | 39 |
| | 52 (31) | 45 (40) |
| | 25 | 35 |
| 4 hours | 32 | 60 |
| | 27 (35) | 42 (45) |
| | 48 | 37 |

* Mice injected with nitrogen mustard on the 5th day of tumor growth, followed by H-3-cytidine or H-3-leucine at the indicated intervals, and killed 20 minutes later.
† As determined by autoradiography on 50 interphase cells per smear.

*Experiment* 5

*Effect of nitrogen mustard on the DNA content of EAT cells.*—The amount of DNA per tumor cell was determined in mice of group 5. The results are shown in Table II, from which it can be seen that, the day after the administration of HN2, the amount of DNA per tumor cell has increased by almost 50 per cent from a mean of 12·2 picograms DNA per cell in control mice, to a mean of 18 picograms DNA per cell in mustard-treated mice. The difference is statistically significant at the 5 per cent level.

TABLE II.—*Effect of Nitrogen Mustard on DNA Content of Ehrlich Ascites Tumor Cells**

| Treatment | EAT cells per c.c. $\times 10^6$ | $\mu$g. DNA per c.c. | Picogram† DNA per cell |
|---|---|---|---|
| HN2 | 59·6 | 1056 | 17·72 |
| HN2 | 63·7 | 1186 | 18·62 |
| HN2 | 45·6 | 812 | 17·80 |
| HN2 | 59·8 | 1072 | 17·92 |
| None | 86·6 | 988 | 11·40 |
| None | 90·2 | 1280 | 14·19 |
| None | 103·4 | 1216 | 11·76 |
| None | 97·2 | 1120 | 11·52 |

* Mice bearing intraperitoneal EAT, injected with 25 $\mu$g. of HN2 on the 4th day of growth and killed 24 hours later.
† Picogram = $10^{-12}$ g.

### DISCUSSION

The life cycle of EAT cells growing in the peritoneal cavity of Strong A male mice has been described in a previous paper (Baserga, 1963), and it has been found to remain fairly constant provided the same sex and strain of mice are used. The entire life cycle covers a period of 18 hours, thus partitioned : $G_1$, less than

1 hour, S phase 10–11 hours, $G_2$ (including the early prophase of Edwards, Koch, Youcis, Freese, Laite and Donalson, 1960) 6 hours, and mitosis, about 1 hour (Hornsey and Howard, 1956). In the first 7 days or so after inoculation, the EAT grows exponentially, with all cells dividing (Edwards *et al.*, 1960 ; Lisco, Nishimura, Baserga and Kisieleski, 1961), while the percentage of cells dying is negligible (Baserga and Gold, 1963). It should be noted that all the experiments described in the present communication were performed in the exponential phase of EAT growth. The decrease in the mitotic index that follows exposure to HN2 indicates that the cells are blocked in the pre-mitotic stage, the $G_2$ phase. The time required for the mitotic index to reach a minimum, 4 hours, suggests that the block takes place or at least is most effective in the first half of the $G_2$ phase. Cells in the latest stages of the $G_2$, probably the early prophase of Edwards *et al.* (1960), may be allowed to reach mitosis, but the flow of cells along the life cycle soon comes to a stop, and the mitotic index drops to a minimum. During this time, RNA and protein synthesis, as evidenced by the uptake of cytidine or leucine, continue at essentially the same rate as in control mice with untreated tumors. The number of cells in DNA synthesis decreases, as shown by the fact that the percentage of tumor cells labeled by a single injection of H-3-thymidine decreases rapidly in the first 8 hours after administration of HN2. However, the uptake of thymidine in those cells still in DNA synthesis is the same as in control animals. This finding suggests that the decrease in the number of cells taking up thymidine is largely due to the continuing flow of cells from the S phase into the $G_2$, at the same time that no new cells are entering in the S phase, because of the block in the $G_2$ stage. These results may explain the discordant data obtained by various authors on the effect of HN2 on nucleic acid synthesis under varying experimental conditions, as reported by Trams, Nadkarni and Smith (1961) and more recently reviewed by Wheeler (1962) and Biesele (1963). The inhibition of DNA synthesis often observed by radiochemical methods may be due to a decrease in the number of cells in DNA synthesis, rather than to a true inhibition of synthesis. The mustard-treated cells not capable of dividing are still able to synthesize DNA as suggested by the fact that the thymidine index rebounds close to normal after about 12 hours, although the mitotic index is still very low. This suggestion is confirmed by the finding that, 24 hours after exposure to nitrogen mustard, the DNA content of mustardized cells is 50 per cent higher than the DNA content of untreated cells. This is in agreement with the findings *in vitro* of Levis, Spanio and De Nadai (1963) who also noticed persistent DNA synthesis in tissue cultures of RCP guinea-pig kidney cells exposed to HN2 even when cell division had almost completely ceased. In addition, these authors showed that RNA and protein syntheses were unaffected, which is in agreement with our own results although, among previous authors, reviewed by Wheeler (1962), nitrogen mustard was found to inhibit synthesis of RNA and proteins in most instances. However, Lee, Richards and Furst (1961) showed an increase in the dry mass of Ehrlich ascites cells, indicative of continued protein synthesis, even after a marked drop in the mitotic index.

Although in previous years DNA has been considered the primary site of attack of alkylating agents (Wheeler, 1962), this hypothesis recently has been challenged, in particular by Rutman, Steele and Price (1961), who found that, in Ehrlich cells exposed *in vitro* to HN2, the proteins bound most of the mustard, and the RNA was also strongly alkylated, while the DNA had only one in every 200,000 deoxynucleotides alkylated. Biesele (1963) felt that this greater alkylation of RNA and proteins was significant, and Levis, Spanio and De Nadai (1963) concluded that mustards acted directly on the division mechanism, inhibition of synthetic processes being secondary. Our experiments using autoradiographic techniques, to analyze the effect of nitrogen mustard on the separate phases of the life cycle of EAT cells growing in the peritoneal cavity of mice, suggest that HN2 may be more effective in inhibiting cell division than in depressing synthetic processes. They indicate that nitrogen mustard acts mostly on EAT cells in the first half of the $G_2$ phase. The effect on DNA synthesis must be limited, at least in time, because the decrease in thymidine uptake can be explained largely as secondary to the pre-mitotic block, and because DNA synthesis resumes even in cells that have not been capable of dividing. However, the results of previous authors together with our own observations indicate that this return to DNA synthesis is largely ineffective, because most of the mustard-treated cells die in the first few days following exposure.

### SUMMARY

Ehrlich ascites tumor cells growing in the peritoneal cavity of mice were exposed to a single injection of nitrogen mustard during the exponential phase of growth. The effect of nitrogen mustard on the life cycle of these cells was investigated by determining the uptake of tritiated precursors by untreated and mustard-treated cells. Nitrogen mustard decreased the number of tumor cells taking up thymidine, but had no immediate effect on the uptake of cytidine and leucine. The mitotic index decreased more rapidly than the thymidine index, and the size of the cells increased with time after exposure to mustard. After 24 hours, the thymidine index had returned close to normal, although the mitotic index was still very low. These findings suggested that nitrogen mustard acts primarily on cells in the pre-mitotic or $G_2$ phase of the life cycle, and that the effect on DNA synthesis is secondary to the blocking of cells in the $G_2$ phase. Cells blocked in the $G_2$ phase eventually go back to synthesizing DNA without prior division. This conclusion was further confirmed by the finding that, in tumor cells 24 hours after exposure to nitrogen mustard, the DNA content per cell was 50 per cent higher than in untreated cells.

This work was made possible by grants from the National Cancer Institute, National Institutes of Health, United States Public Health Service, and from the Illinois Division of the American Cancer Society. One of the authors (R. B.) is a U.S.P.H.S. Research Career Development Awardee.

### REFERENCES

Baserga, R.—(1963) *Arch. Path.*, **75**, 156.
*Idem* and Gold, R.—(1963) *Exp. Cell Res.*, **31**, 576.
*Idem* and Kisieleski, W. E.—(1962) *Atompraxis*, **8**, 386.
Biesele, J. J.—(1958) ' Mitotic Poisons and the Cancer Problem ', Amsterdam (Elsevier Publishing Company).—(1963) *Exp. Cell Res.* (suppl.), **9**, 525.
Drysdale, R. B., Hopkins, A., Thomson, R. Y., Smellie, R. M. S. and Davidson, J. N.—(1958) *Brit. J. Cancer*, **12**, 137.
Edwards, J. L., Koch, A. L., Youcis, P., Freese, H. L., Laite, M. B. and Donalson, J. T.—(1960) *J. biophys. biochem. Cytol.*, **7**, 273.

FRIEDENWALD, J. S.—(1951) *Ann. N.Y. Acad. Sci.*, **51**, 1432.
HORNSEY, S. and HOWARD, A.—(1956) *Ibid.*, **6**, 915.
HOWARD, A. AND PELC, S. R.—(1953) *Heredity* (suppl.), **6**, 261.
HUGHES, A. F. W.—(1950) *Quart. J. micr. Sci.*, **91**, 251.
JOFTES, D. L. AND WARREN, S.—(1955) *J. biol. photogr. Ass.*, **23**, 145.
LAJTHA, L. G.—(1957) *Physiol. Rev.*, **37**, 50.
LEE, H., RICHARDS, V. AND FURST, A.—(1961) *Cancer Res.*, **21**, 1108.
LEVIS, A. G., SPANIO, L. AND DE NADAI, A.—(1963) *Exp. Cell Res.*, **31**, 19.
LISCO, H., NISHIMURA, E. T., BASERGA, R. AND KISIELESKI, W. E.—(1961) *Lab. Invest.*, **10**, 435.
MESSIER, B. AND LEBLOND, C. P.—(1957) *Proc. Soc. exp. Biol. N.Y.*, **96**, 7.
RUTMAN, R. J., STEELE, W. J. AND PRICE, C. C.—(1961) *Cancer Res.*, **21**, 1134.
SCOTT, J. F., FRACCASTORO, A. P. AND TAFT, E. B.—(1956) *J. Histochem. Cytochem.*, **4**, 1.
SUGIURA, K.—(1953) *Cancer Res.*, **13**, 431.
TRAMS, E. G., NADKARNI, M. V. AND SMITH, P. K.—(1961) *Ibid.*, **21**, 567.
WHEELER, G. P.—(1962) *Ibid.*, **22**, 651.